# Shedding Lights on Crude Venom from Solitary Foraging Predatory Ant *Ectatomma opaciventre*: Initial Toxinological Investigation

**DOI:** 10.3390/toxins14010037

**Published:** 2022-01-04

**Authors:** Lucas Ian Veloso Correia, Fernanda Van Petten de Vasconcelos Azevedo, Fernanda Gobbi Amorim, Sarah Natalie Cirilo Gimenes, Lorena Polloni, Mariana Alves Pereira Zoia, Mônica Soares Costa, Jéssica Peixoto Rodrigues, Kelly A. Geraldo Yoneyama, Jean Carlos Santos, Eliane Candiani Arantes, Veridiana de Melo Rodrigues, Luiz Ricardo Goulart, Renata Santos Rodrigues

**Affiliations:** 1 Laboratory of Biochemistry and Animal Toxins, Institute of Biotechnology, Federal University of Uberlândia, Uberlândia 38408-100, MG, Brazil; fvpetten@yahoo.com (F.V.P.d.V.A.); sarah_gi_menes@hotmail.com (S.N.C.G.); polloni.lorena@gmail.com (L.P.); monicacosta.farma@gmail.com (M.S.C.); kellyagy@gmail.com (K.A.G.Y.); vmravila@hotmail.com (V.d.M.R.); 2 Laboratory of Nanobiotechnology Institute of Biotechnology, Federal University of Uberlândia, Uberlândia 38408-100, MG, Brazil; marianazoia@hotmail.com (M.A.P.Z.); jessica.prt@hotmail.com (J.P.R.); goulartlr@gmail.com (L.R.G.); 3 Laboratory of Animal Toxins, Faculty of Pharmaceutical Sciences of Ribeirão Preto, Universidade de São Paulo, Ribeirão Preto 14040-900, SP, Brazil; fernandagamorim@gmail.com (F.G.A.); ecabraga@fcfrp.usp.br (E.C.A.); 4 Laboratory of Immunopathology, Institute Butantan, São Paulo 05503-900, SP, Brazil; 5 Department of Ecology, Institute of Biology, Federal University of Sergipe, São Cristovão 49060-108, SE, Brazil; jcsantosbio@gmail.com

**Keywords:** ant venom, *Ectatomma opaciventre*, enzymatic assays, antitumoral activity, lung cancer, antiparasitic effects, *Leishmania*, hyaluronidase, L-amino acid oxidase

## Abstract

Some species of primitive predatory ants, despite living in a colony, exercise their hunting collection strategy individually; their venom is painful, paralyzing, digestive, and lethal for their prey, yet the toxins responsible for these effects are poorly known. *Ectatomma opaciventre* is a previously unrecorded solitary hunting ant from the Brazilian Cerrado. To overcome this hindrance, the present study performed the in vitro enzymatic, biochemical, and biological activities of *E. opaciventre* to better understand the properties of this venom. Its venom showed several proteins with masses ranging from 1–116 kDa, highlighting the complexity of this venom. Compounds with high enzymatic activity were described, elucidating different enzyme classes present in the venom, with the presence of the first L-amino acid oxidase in Hymenoptera venoms being reported. Its crude venom contributes to a state of blood incoagulability, acting on primary hemostasis, inhibiting collagen-induced platelet aggregation, and operating on the fibrinolysis of loose red clots. Furthermore, the *E. opaciventre* venom preferentially induced cytotoxic effects on lung cancer cell lines and three different species of *Leishmania*. These data shed a comprehensive portrait of enzymatic components, biochemical and biological effects in vitro, opening perspectives for bio-pharmacological application of *E. opaciventre* venom molecules.

## 1. Introduction

Insects are invertebrates with a wide variety of defense mechanisms, which is why they have managed to occupy the most diverse terrestrial environments [1]. Ants are aculeated Hymenoptera, as they have an inoculation device (stinger), in addition to the corresponding secretory glands, where the venom is produced, stored in a reservoir connected to the inoculation device [2,3]. From 21 subfamilies of Formicidae, five lost their sting in the evolutionary process, through changes in the morphology of the terminal region of the gaster (such as the acidopore of the subfamily Formicinae, used to project a toxic composed basically of formic acid and hydrocarbons). Thus, 16 subfamilies retained the ability to use their venom to search for food by subduing their prey [4]. This foraging and predation habit is exercised collectively or individually, with the latter group standing out for having a sufficiently powerful venom to incapacitate their prey [5].

Although ant venoms are richly diverse in toxins, bioactive compounds, alkaloids, proteins, and peptides [4,6,7,8], knowledge about the toxinology of these animals remains limited or even largely unknown due extensively to the number of species. The lack of information about the effects derived from crude venom stands out in this scenario, as such understanding is important from a medical point of view in cases of allergies, envenoming, and anaphylaxis, as well as from the perspective of these effects in the process of drug discovery [9,10,11]; this is exemplified by the most famous success of a toxin that bioinspired the design of Captopril, from the observation of the hypotensive effect of the bite by the Brazilian snake *Bothrops jararaca* [11].

This lack of data in ants is mainly due to the limited amount of venom that can be obtained from a single ant as well as the laborious dissection and extraction of the venom, especially in solitary foraging ants, as the capture is completed individually, instead of by entire colonies, like ants as previously reported in the *Solenopsis* genus [12]. Despite the limitations, interest in ant venoms has increased in recent years with interesting actions being reported as antiparasitic, antimicrobial, and antinociceptive action on *Dinoponera quadriceps* venom [13,14,15], insecticidal activity on *Manica rubida* venom [16], and modulation of immune response and redox balance in vivo by the *Brachyponera sennaarensis* venom [17].

The genus *Ectatomma* (Hymenoptera: Formicidae: *Ectatomminae*) has 14 described species present in the Neotropical region and, despite living in colonies, they exercise their foraging and predation solitarily [18]. In 1994, Arseniev et al. described for the first time a component from *Ectatomma tuberculatum* venom, ectatomin, a hetorodimeric neuropeptide acting on calcium channels and forming pores in the membrane cells [19,20]. Twenty years later, synthetic peptides homologous to *Ectatomma quadridens* [homonym replaced by *Ectatomma brunneum*] venom showed antimicrobial and cytotoxic activity against tumor cells [21]. Thus, solitary foraging ants, mainly of the *Ectatomma* genus, present a large field of research due to the lack of studies on the characterization of the crude venom and the low number of isolated components. *Ectatomma opaciventre* is a relatively large predatory ant (1.5 cm) that exhibits well-developed mandibles and a stinger apparatus; its geographic distribution covers some countries in South America, Brazil being the largest record of the presence of the species [18].

Given the above, the characterization of the venom of ant species can contribute to a better understanding of the evolution of ant and Hymenoptera toxins, as well as the variability of the venoms within the venomous ant genera and subfamilies, toxic potential to man, and important biological tool potential in drug discovery. Herein, we intend to better understand the venom of the solitary foraging ants *E. opaciventre*, through a descriptive approach of their biochemical effects, the activity and description of the enzyme classes present in the venom, and the biotechnological potentials and therapeutic applications of the venom.

## 2. Results

### 2.1. Analysis of Peptides, Proteins, and Zymography Profile in Polyacrylamide Gel from E. opaciventre Venom

**Figure 1:**
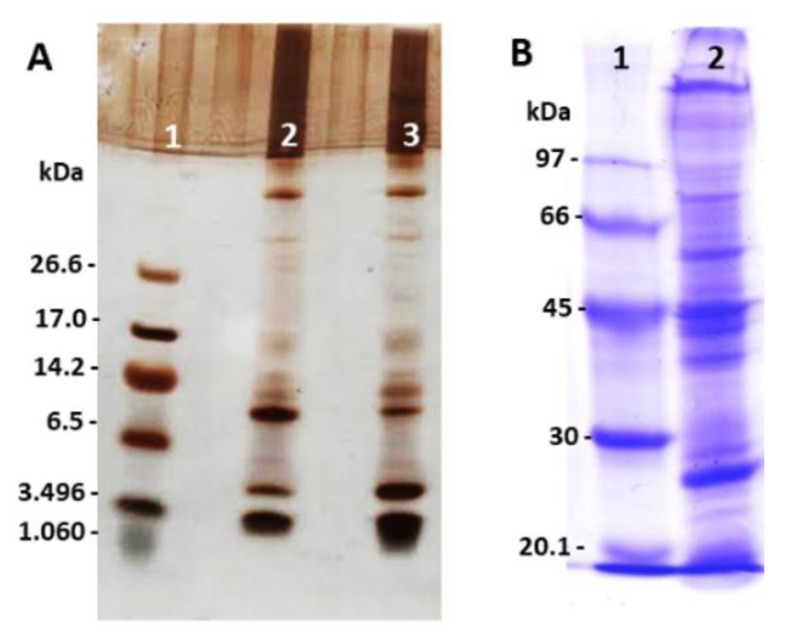
Protein profile by SDS-PAGE. (**A**) SDS-PAGE (21%) stained with silver. (1) Molecular Mass Standard. (2) 40 μg of the crude venom of *E. opaciventre* (non-reduced). (3) 40 μg of the crude venom of *E. opaciventre* (reduced by β-mercaptoethanol). (**B**) SDS-PAGE (12.5%) stained with comassie blue. (1) Molecular Mass Standard. (2) 40 μg of the crude venom of *E. opaciventre* under reduced conditions.

The ant venoms showed great variability in their composition, with a large protein range from 1 kDa to 114 kDa. SDS-PAGE analysis demonstrated the diversity of components among proteins and peptides in *E. opaciventre* venom, with intense bands of peptides (Figure 1A) and proteins (Figure 1B). Figure 1A shows some differences between reduced and non-reduced profiles, especially at 6.5 and 3.49 kDa. These results suggest the presence of proteins in different state of oligomerization. The non-reduced sample shows an intense band around 6.5 kDa and less intensity on the reduced sample at the same molecular mass; the opposite can be observed around 3.49 kDa, where the reduced sample has a more intense band and less intensity on the non-reduced sample. In this context, we can exemplify the presence of peptides on dimers conformation on the venom composition.

**Figure 2:**
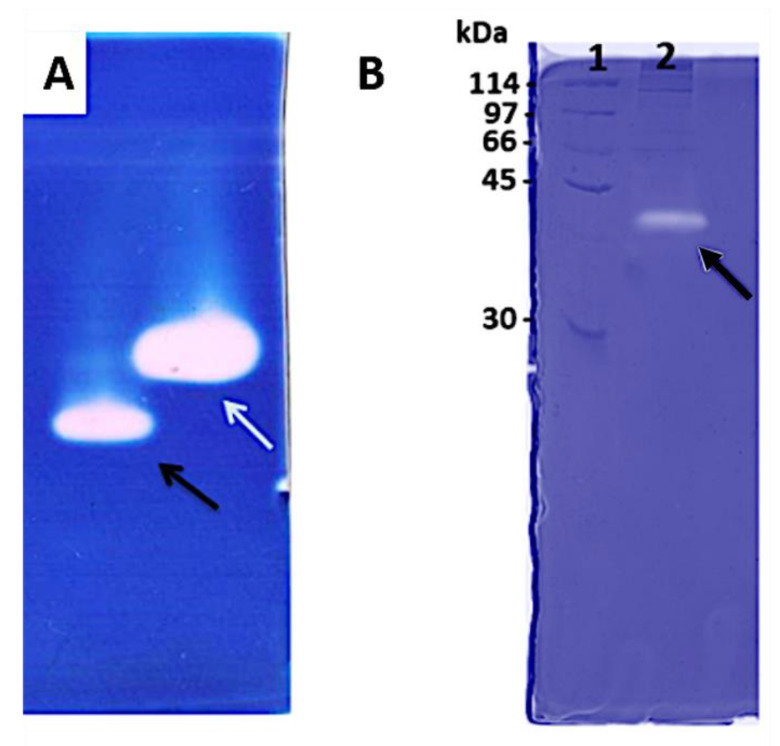
Zymography assay. (**A**) Acrylamide-hyaluronan zymography gel from the crude venom of *E. opaciventre*, analyzed under non-reduced conditions. (1) 40 μg of the crude venom of *E. opaciventre*; the black arrow shows the protein activity around 38 KDa. (2) Sample of scorpion *Tityus serrulatus* as a positive control; the white arrow shows the protein activity around 40 KDa. (**B**) Acrylamide gelatin-zymogram under non-reduced conditions of the crude venom of *E. opaciventre*. (1) Molecular Mass Standard. (2) 10 μg of the crude venom of *E. opaciventre*.

When we analyzed the proteolytic capacity of venom proteins, the zymography method was able to associate the nature and molecular mass of the diverse proteins present in the venom, highlighted by their intense enzymatic performance. Gelatin and hyaluronan were also used for copolymerization of the acrylamide gel to show the enzymatic activity in a different substrate. The results showed a band of approximately 38 kDa evidencing high hyaluronidase activity (Figure 2A), while a proteolytic activity was observed in the 40 kDa range when gelatin was topped as a substrate (Figure 2B). All these results together showed a rich variety of proteolytic compounds on *E. opaciventre* venom.

### 2.2. Detection of Enzyme Classes Contained in the E. opaciventre Crude Venom and Quantitative Evaluation of Activity

**Figure 3:**
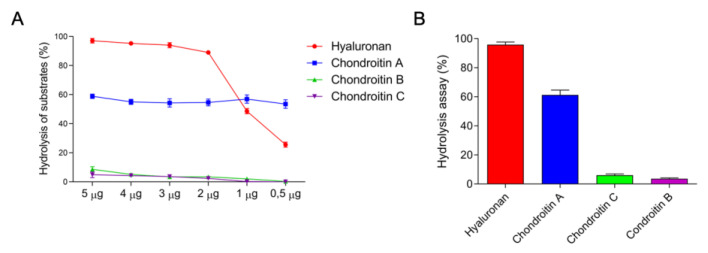
Hyaluronidase activity profile. (**A**) Turbidimetric activity of the different concentration of crude venom, from *E. opaciventre* on different substrates that were incubated for 30 min in 200 mm acetate buffer, 150 mm sodium chloride pH 6. The hydrolysis reaction was quenched with 5% BCTA and 4 NaOH% (*w*/*v*). The activity was read at 400 nm and expressed as a percentage. All data are expressed as mean ± S.E.M and procedures were carried out in triplicate. (**B**) The preferential degradation profile of hyaluronidases from *E. opaciventre* venom.

Due to the large diversity of the protein bands on SDS-PAGE and the identification of the enzymatic activity of some components of the venom, showed in Figure 2, a deeper characterization of these components was performed. Hyaluronidase activity demonstrated that 1.12 μg were required to degrade 50% of the hyaluronan present in the medium, thereby obtaining 892.65 UTR/mg of specific activity (Figure 3A). The enzymes present in the venom showed a preference for degrading hyaluronan, followed by chondroitin sulfate A, C, and B, hydrolyzing about 98%, 55%, 19%, and 12% of the substrates contained in the medium, respectively (Figure 3B).

**Figure 4:**
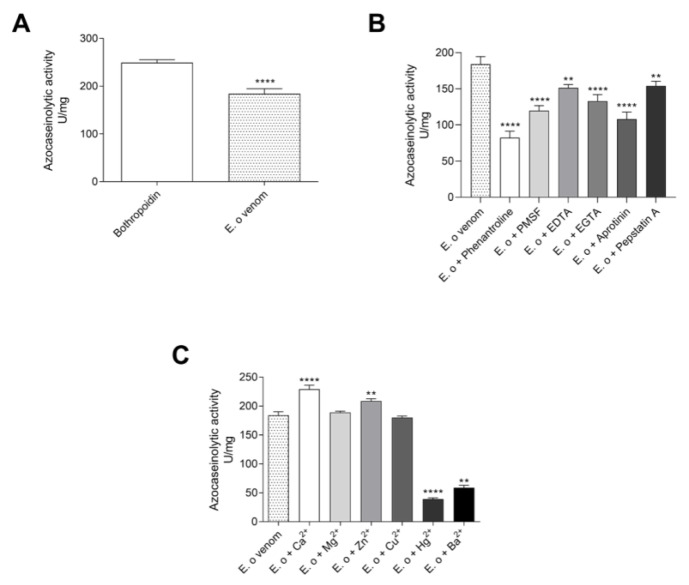
Protease characterization assays. (**A**) Proteolytic activity of the crude venom on azocasein; 5 μg of *E. opaciventre* ant venom and 5 μg of Bothropoidin, a metalloprotease purified from *Bothrops pauloensis* venom (positive control), incubated for 30 min at 37 °C. (**B**) Influence of different inhibitors at a concentration of 10 mM, previously incubated with 5 μg of *E. opaciventre* ant venom for 30 min. (**C**) Influence of different divalent cations at a concentration of 10 mM, previously incubated with 5 μg of *E. opaciventre* ant venom for 30 min. The reaction was quenched with addition of 20% trichloroacetic acid (*v*/*v*). The activity was read at 405 nm and expressed as U/mg. All data are expressed as mean ± S.E.M and procedures were carried out in triplicate. Statistically significant differences in relation to control (*E. opaciventre* venom). ** (*p* ≤ 0.01) and **** (*p* ≤ 0.0001).

The azocaseinolytic activity of *E. opaciventre* crude venom was of 184 U/mg (Figure 4A). This value was similar to the positive control known by higher azocaseinolytic activity, the Metalloproteinase Bothropoidin from *Bothrops pauloensis* venom [22]. In addition, to investigate the effect of inhibitory agents on the proteolytic activity of the proteins’ venom, we performed the azocaseinolytic activity after venom incubation with different chemical agents (Figure 4B). When we analyzed all inhibitors tested, the 1,10-phenanthroline and Aprotinin were able to reduce the venom activity on the degradation of the substrate and, in a lower capacity, the Pepstatin A. The 1,10-phenantroline showed maximum inhibition of 55%, followed by Aprotinin (41%), PMSF (35%), EGTA (27%), EDTA (17%), and Pepstatin A (16%), when compared to the activity of the crude venom (positive control). The azocasein substrate assay was also performed in the presence of divalent cations (Ca^2+^, Mg^2+^, Zn^2+^, Cu^2+^, Hg^2+^ and Ba^2+^). Our results showed that Ca^2+^ and Zn^2+^ were able to slightly increase the proteolytic activity, with Ca^2+^ being the most active. These results could suggest a higher presence of enzymes Ca^2+^ and Zn^2+^, depending (Figure 4C). However, the Hg^2+^ and Ba^2+^ caused a stronger reduction in the proteolysis of the substrate, which suggests a possible enzymatic inhibition due to the competition of ions.

**Figure 5:**
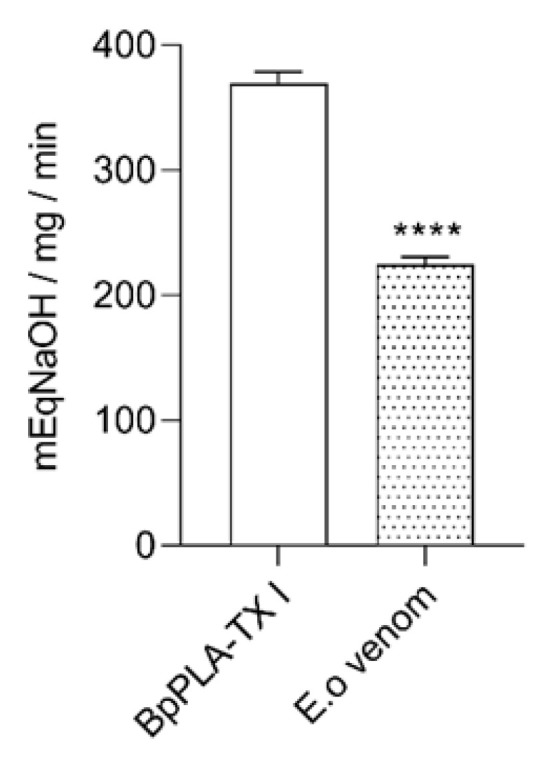
Phospholipase detection. Hydrolysis of phospholipids by the action of 5 μg of crude venom from *E. opaciventre* and 5 μg of BpPLA_2_ TX-I (positive control). Phospholipase activity was monitored with a pH meter for 3 min. The activity was expressed in mEqNaOH/mg/min. Data show the mean ± standard deviation (S.D.) Statistically significant differences in relation to control (BpPLA_2_-TXI). **** (*p* ≤ 0.0001).

Following the functional characterization, the phospholipase activity was also performed by the capability of hydrolysis of phospholipids through egg yolk methodology. The halos generated by the indirect activity in agar containing egg yolk for 24 h of incubation with the venom were 2.8 cm, thus demonstrating a high phospholipase activity (data not shown). The indirect activity does not show the specific phospholipase activity. In order to evaluate the specific activity of phospholipase present on *E. opaciventre* venom, we also investigated the substrate hydrolysis assay, a direct phospholipase A_2_ activity. This assay was performed by a pH meter, and the value of *E. opaciventre* venom activity determined was 222 mEqNaOH/mg/min (Figure 5). In comparison with the positive control, a phospholipase A_2_ isolated from *B. pauloensis* with an intense enzymatic activity [23], the *E. opaciventre* showed a greater phospholipase activity. These results suggest the presence of Phospholipase A_2_, in an active state known as Asp-49 Phospholipase A_2_.

Going forward on *E. opaciventre* venom characterization, we also performed tests to investigate the presence of L-amino acid oxidases (LAAOs). LAAOs is an important group of toxins mainly described in snake venom. Our group has been working on snake venom characterization and described the presence of LAAOs in different snake venom. Using this background of knowledge, we used L-amino acids as a substrate for oxidative determination to determine the presence of LAAOs on *E. opaciventre* venom (Table 1). In this context, our results showed a maximum activity of 384.18 U/mg for serine and a minimum activity of 153.2 U/mg for asparagine, as well as the average value of 310.43 U/mg for Valine, 290.05 U/mg for Glutamine, 259.49 U/mg for Alanine, and 161.48 U/mg for Arginine. When we compared these results with data collected before by our group from *B. pauloensis* venom, the *E. opaciventre* venom showed higher activity, especially for Serine, which had no activity on snake venom, Valine with 110 U/mg, Glutamine with 140 U/mg, Alanine with 60 U/mg, and 340 U/mg for Arginine [24]. The function of LAAOs on ant venom remain uncertain; however, our data suggest, at this point, a rich composition of LAAOs with intense activity. Moreover, all enzymatic description together highlights the large variability of venom components present in *E. opaciventre* venom.

### 2.3. Evaluation of Effects upon Biochemical Parameters Por E. opaciventre Venom

**Figure 6:**
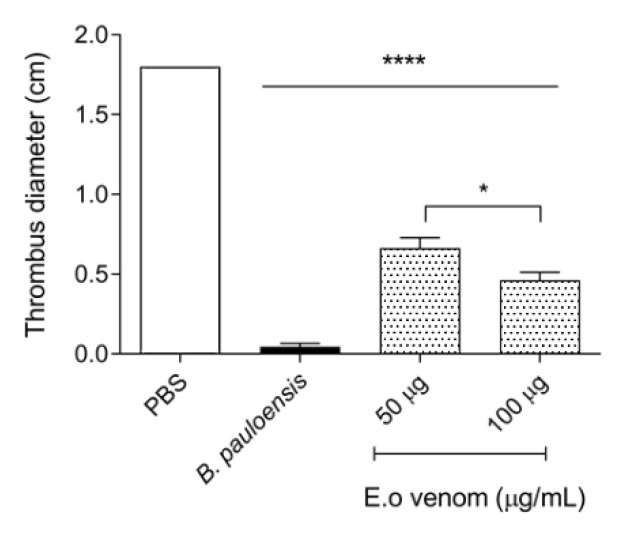
Thrombolytic activity on red clots incubated at 37 °C for 48 h. The samples of *E. opaciventre* venom were prepared in phosphate buffered saline (PBS). As controls, 50 μg of the *Bothrops pauloensis* snake venom was used. Data show the mean ± standard deviation (S.D.) Statistically significant differences in relation to control, positive control by *B. pauloensis* venom, and phosphate saline buffer as negative control * (*p* ≤ 0.05) and **** (*p* ≤ 0.0001).

As we showed before, *E. opaciventre* venom has a pronounced proteolytic enzyme content that can act on hemostasis parameters. In this context, we have performed some of the key assays by which these enzymes can operate. The venom did not exert fibrinogenolytic activity when incubated at different concentrations with bovine fibrinogen for 2 h, verified by SDS-PAGE (data not shown); likewise, when evaluated, the venom was not able to coagulate the plasma in a time less than 240 s (data not shown). All information suggests that the *E. opaciventre* venom has no effect on hemostasis disorder. However, the *E. opaciventre* venom performed a remarkable action by degrading about 50% of the spontaneous clots with 48 h of incubation (Figure 6). Even though, when we compared with the positive control *Bothrops pauloensis* venom (described as a potent thrombolytic venom), our results showed a reasonable thrombolytic. This result was important to suggest a great capacity and potential properties of thrombolysis agent.

### 2.4. Inhibition of Platelet Aggregation by E. opaciventre Venom

**Figure 7:**
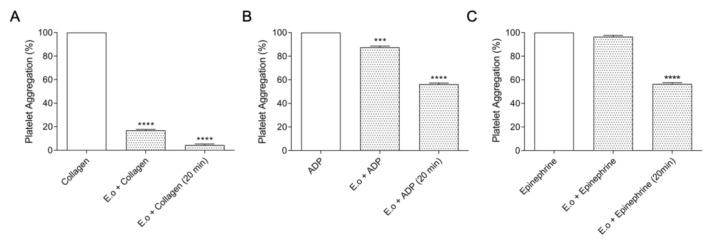
Effects of *E. opaciventre* crude venom on platelet aggregation. Activation of platelet aggregation (3–4 × 10^5^/μL) induced by different agonists: adenosine diphosphate, ADP (20 mm); Collagen (100 μg/mL) and Epinephrine (300 μM/L). Platelet aggregation was recorded for 10 min in AggRAM platelet aggregation system with four-channel laser optics (Helena Laboratories, TX, USA) adding the agonist and the venom simultaneously at the time of testing or pre-treating the platelet solution for 20 min with the *E. opaciventre* venom. A representative graph of platelet aggregation activity. (**A**) Collagen (100 μg/mL). (**B**) Adenosine diphosphate, ADP (20 mm). (**C**) Epinephrine (300 μM/L. Statistically significant differences regarding ADP, Collagen, and Epinephrine as positive control. All data are expressed as mean ± S.E.M and procedures were carried out in triplicate; statistically significant, *** (*p* ≤ 0.001) and **** (*p* ≤ 0.0001), treatments compared to control.

Following the functional characterization, we investigated the capacity to inhibit the platelet aggregation. Platelets play a key role in primary hemostasis by contributing to the membrane receptors that facilitate and amplify the localized activity of blood clotting factors. This assay characterizes the interference of *E. opaciventre* crude venom (30 µg) in platelet aggregation induced by agonists (ADP, collagen and epinephrine) (Figure 7). The results showed that crude venom strongly inhibits collagen-induced aggregation in 81.4% (Figure 7A), when compared to ADP 12.5% (Figure 7B) and epinephrine 1.22% (Figure 7C). When evaluated for aggregation with platelets previously treated for 20 min with the crude venom, the data reveal an inhibition of 94.19% when mediated by collagen, followed by 45.56% by epinephrine and 43.25% by ADP.

### 2.5. Bioprospecting Characterization

#### 2.5.1. Evaluation of the Toxicity by *E. opaciventre* Venom on Lung Cancer Cells

**Figure 8:**
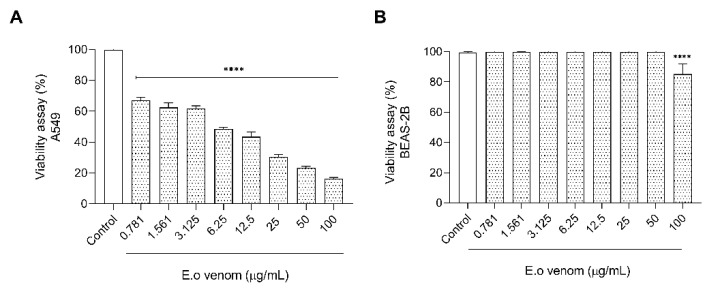
Graphic of viability of tumorigenic and non-tumorigenic cell lines. Cells were cultured in 96-well plates in the absence (control) or presence of different concentrations of the *E. opaciventre* venom. (**A**) Effects on Lung Cancer (A549) cell viability induced by *E. opaciventre* venom. (**B**) Graph represents the viability of Epithelial Lung (BEAS-2B) cell lineage treated with *E. opaciventre* venom. The viability is analyzed by an MTT assay. Data show the mean ± standard deviation (S.D.). Statistically significant difference **** (*p* < 0.001) compared with control (BEAS-2B).

After all functional characterization and the greatest variability of protein compounds on *Ectatomma opaciventre* venom, we started an investigation into the potential presence of biomolecules for bioprospection. Since the discovery and production of biomimetic drugs from animal toxins, great attention has been shown to these natural libraries of bioactive molecules in the bioprospecting scenario. In this context, *Ectatomma opaciventre* venom demonstrated cytotoxicity against A549 cells (Figure 8) via an MTT assay. The venom was able to reduce the cell viability in a concentration-dependent manner, inducing death at a range of approximately 83% of the highest concentration tested (100 µg/mL). It is important to highlight that the venom did not significantly affect the death of a non-tumorigenic bronchus cell lineage, BEAS-2B, suggesting a specific action on tumorigenic cells (A549).

#### 2.5.2. Cytotoxic Effect of *E. opaciventre* Venom on Promastigote Forms of Leishmania

**Figure 9:**
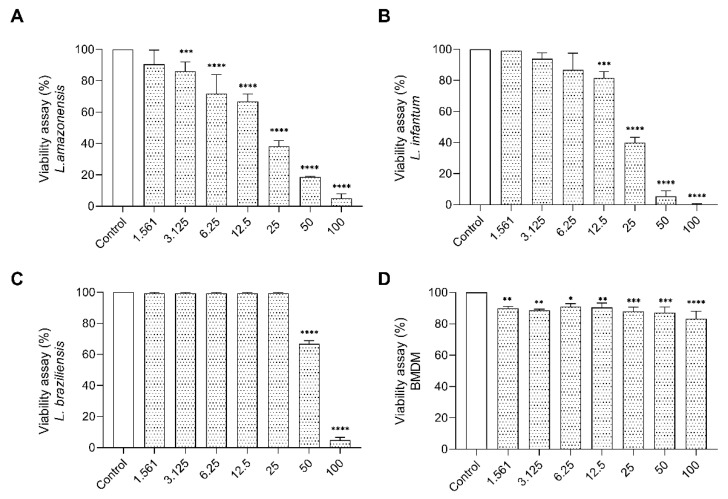
Antiparasitic Assay. Effects of *E. opaciventre* ant venom on the viability of different parasites. The viability of parasites cultured for 72 h in the presence of different concentrations of the venom and absence (control), analyzed by the MTT colorimetric method. (**A**) Viability on the promastigote form of *L. amazonensis* treated with *E. opaciventre* venom. (**B**) *L. braziliensis* viability treated with *E. opaciventre* venom. (**C**) Analysis of *L. infantum* viability after treatment with *E.* opaciventre venom. (**D**) BMDM cell viability after exposure to *E. opaciventre* venom. Data show the mean ± standard deviation (S.D.). Statistically significant difference * (*p* < 0.05), ** (*p* < 0.01), *** (*p* < 0.001) and **** (*p* ≤ 0.0001) compared with control.

Going forward with bioprospecting characterization, we performed the cytotoxic assay on promastigote forms of *Leishmania.* The treatment of promastigote with the *E. opaciventre* venom showed significant inhibition in viability of parasites. The data obtained at 72 h allowed us to estimate an IC_50_ of 50.98 μg/mL to *L. braziliensis*, 28.00 μg/mL to *L. amazonensis*, and 22.30 μg/mL to *L. infantum*. We also observed that the morphological alterations on treated parasites were a rounded and unusual cell body, with loss of membrane integrity, corroborated by 100% parasite death when treated with the highest dose against *L. infantum*, 96.6% against *L. braziliensis*, and 95.1% against *L. amazonensis* (Figure 9). To enhance the bioprospecting properties of *E. opaciventre* venom, we verified the potential cytotoxicity on macrophage cells. Bone marrow-derived macrophage (BMDM) cells were used as a control, since they are the main cell line involved on the parasitic infection pathway. Thus, when compared with BMDM cells, the *E.* *opaciventre* venom did not evoke a significant toxic response, triggering a decrease in viability of around 10% at the high concentration tested (Figure 9). These results suggest a preference action for promastigote forms rather than normal cells, which is a crucial point for developing new drug designs.

## 3. Discussion

Many of the venoms from the most diverse animal species are a complex mixture of bioactive components, such as proteins, peptides, and organic and inorganic components such as biogenic amines and neurotransmitters [4,6,7,8]. In the animal kingdom, venom has several functions, the most important are defense against predators and capture of prey [25]. Solitary foraging ants need venom sufficiently powerful to individually deal with the defense and capture of their prey, making their venom an interesting target for study [5,18].

Despite advances promoted by venomics (represented by mass spectrometry in proteomic and peptidomic studies and by molecular biology in transcriptomic approaches to venoms) allowing for a deep knowledge of the ant venoms’ composition [26,27,28,29], the effects of crude venom or isolated toxins are untapped, fostering different hypotheses about the role of venom, the biochemical, biological, and clinical implications, and in the process of drug discovery [7,8,11]. For example, several studies have already approached ant venom through venomics tools [28,30,31,32,33,34,35], and many reviews have highlighted the importance of the allergic response to envenomation by ants [10,36,37,38,39]. Nevertheless, this significant biomedical interest remained poorly characterized until 2018, when Zamith-Miranda and collaborators cast a new light on the mechanisms of the allergenic process mediated by a proteinaceous extract from fire ants [40].

Due to the aforementioned, it is necessary whenever possible, given the difficulties of obtaining a sample (already highlighted) and the huge number of species, to carry out studies that provide knowledge not only in “omic” manner, but also regarding the function of crude venom and toxins. This comprehensive portrait will enable a holistic knowledge in the toxinological, ecological, evolutionary, and clinical aspects of ants, as well as providing new insights into bioprospection of biomolecules with potential biopharmacological action.

### 3.1. Evaluation of Enzymatic Profile from E. opaciventre Venom

This is the first exploratory study of the biological action of the solitary predator ant *Ectatomma opaciventre* venom. Analysis of the electrophoretic profile of the venom showed intense bands of low molecular weight components in the range of 1.5 kDa, 4.5 kDa and 10 kDa. Some differences between the molecular signature between the reduced and non-reduced profile by β-mercaptoethanol, which occupies the presence of dimeric polypeptides in the venom of *Ectatomma opaciventre*. It is already known that polypeptides found in ant venoms can be structurally classified as linear, dimeric, and inhibitors of the cystine node (ICK-like) [4].

Hymenopteran venoms contain a large diversity of peptides that constitute a predominant class of toxins in ant venoms [4,21,41,42]. Despite being extensively studied for their biological activity, the envenoming effects of ant peptides are still poorly understood. Ectatomin, a neuropeptide isolated from *Ectatomma tuberculatum* venom, has a molecular mass of 7.8 kDa and is composed of two subunits held together by a bound disulfide. Ectatomin is a potent inhibitor of calcium channels, acting in isolated rat myocardium, as reported by Pluzhni-kov et al. (1999) [19,20,43]; our data revealed the presence of dimeric peptides by SDS-PAGE analysis of the venom of *E. opaciventre*. Regarding the components of high molecular mass, our results revealed intense protein bands distributed from 14.4 kDa to 114 kDa. The knowledge accumulated in the field of toxinology to date about toxins from animal venoms, allows us to suggest the presence of some classes of enzymes based on their molecular mass, combined with the functional characterization performed [6,7,44]. The enzymatic characterization pointed out some of the main classes of enzymes from animal venoms present in *E. opaciventre* venom.

First, two distinct enzymatic activities were demonstrated by the *E. opaciventre* venom using a zymogram technique co-polymerized with hyaluronan and gelatin, thus enabling the presence of hyaluronidases and proteases in the venom. Moreover, when we combined the results from the influence of different divalent cations on enzymatic activity, we can also suggest the presence of an ion-dependent enzyme, such as metalloproteinase. In agreement with our results, several proteases were identified and characterized in the crude venom of the ant *Odontomachus bauri* by collagen zymogram assay [45]. In addition, hyaluronidases from invertebrate and vertebrate venom animals were already described using polyacrylamide gel co-polymerized with hyaluronan [46], which corroborate with our data and conclusions.

#### 3.1.1. Hyaluronidases

Hyaluronidases are the group of enzymes that preferentially cleave hyaluronan, also being capable of degrading chondroitin and chondroitin sulfate to a lesser degree. These glycosaminoglycans are present in the extracellular matrix (ECM) of different organisms [44]. Thus, when degrading the components of the interstitial matrix, the animal venom hyaluronidases are considered spreading factors, aiding and enhancing the diffusion of toxins in the tissue/organism of the prey/victim. In Hymenoptera, these enzymes also can act as allergens, being able to induce anaphylactic shock mediated by IgE, which can be fatal in some cases [30,44,47,48]. In *E. opaciventre*, after detecting the presence of this class of enzymes by zymogram, we analyzed the specific activity under hyaluronan substrate, but also of the main chondroitin present in the ECM. This approach allowed us not only to quantify the specific activity, but also to characterize the degradation profile of the hyaluronidases contained in the venom, highlighting the potency of this class of enzymes.

The presence of hyaluronidases has already been reported in the genome of *Solenopis invicta* [49] and *Saltator harpegnatos* [50] species; at the protein level, hyaluronidase fragments were detected by bottom-up proteomic techniques in the *Dinoponera quadriceps* [51] and *Solenopsis invicta* [52] venoms and top-down in the *Pachycondila striata* [53] and *Paraponera clavata* [30,31] venoms. The hyaluronidase activity has already been registered in the venoms of *Pogonomyrmex baldius*, *Ectatomma tubeculatum*, and *Odontomachus haematodus* [54]; unfortunately, the protocol differences between them make it impossible to compare the activity quantitatively. Meanwhile, the hyaluronidase activity of the *E. opaciventre* venom was 892.65 UTR/mg, which is six times more active than the enzyme from *Crotalus durrissus terrificus* venom that hydrolyzes hyaluronan, following the same experimental procedure [55], which emphasizes the effectiveness of this enzyme class on venom composition. It is important to highlight that our study was the first to trace a hyaluronan degradation profile of ant venoms, increasing the knowledge of this enzyme class in ant venom species.

#### 3.1.2. Proteases

In general, venom proteases are involved in processes of necrosis, inflammation, edema, tissue damage, disturbance of the hemostatic system, modulation of defense mechanisms, and in the spread of venom in victims/prey. Classically, proteases are also credited with the biological role of initiating pre-digestion as well as for their ability to cleave peptide bonds, thus making prey more digestible [52,56]. The presence of metalloprotease in the venom of *Sonenopsis invicta* has been reported by proteomic approaches [52]. Schimidt et al. reported very high proteolytic activity in *Eciton burchelli* [54]. An interesting finding was revealed by a recombinant serine protease from *Scleroderma guani* venom with low trypsin-like catalytic activity; however, in biological tests in *Tenebrio molitor* larvae, this enzyme was able to inactivate the polyphenoloxidase enzyme that plays a key role in the immune cascade response of insects [57]. Furthermore, Hoffman et al. demonstrated that Hymenoptera proteases are also important allergic mediators with a high capacity to bind immunoglobulins IgE [58,59].

Initially, the ability to degrade a protein substrate was evaluated in a collagen copolymerized zymogram. Then, a quantitative analysis under the substrate azocasein demonstrated a potent proteolytic activity of the *E. opaciventre* venom. Our data reveal that the chelating activity of the phenanthroline, EGTA, and EDTA inhibitors is critical for part of the enzyme’s components in the venom. The presence of proteases from *Odontomachus bauri* crude venom exerted an optimal activity at pH 8.0 at 37 °C on azocasein substrate, while in the presence of inhibitors the proteolytic activity was significantly reduced: aprotinin (45%), aprotinin (25%) and EDTA (9%) [45]. In contrast to our data, the *Odontomachus bauri* venom did not show any influence of divalent cations tested on proteolytic activity.

Another fraction of significantly inhibited proteases occurred when the crude venom was previously treated with PMSF and aprotinin. PMSF and aprotinin are general inhibitors of serine proteases that react with serine residues to inhibit the catalytic site of enzymes such as trypsin, chymotrypsin, and thrombin, indicating the presence of this class in *E. opaciventre* venom. Whitworth and coworkers isolated and characterized the presence of four serine proteases from *Solenopsis invicta* larvae that were shown to be inhibited by diisopropyl fluorophosphate, an inhibitor of serine proteases [60]. Following this context, we can see that the results are highlighted by the increasement on enzymatic activity when the venom was previously incubated with Ca^2+^ and Zn^2+^ ions. It suggests that these cations are the main cofactors of the proteases present in the venom. Taken together, these results point to the presence of metalloproteases in the venom of *Ectatomma opaciventre,* which is already known for their presence in different animal venom and for cofactor Zn^2+^ [22].

The proteolytic activity in the presence of Hg^2+^ and Ba^2+^ was slightly inhibited, and the interference of metal ions in the proteolytic activity is widely described for venom metalloproteases, since the binding of these ions can lead to a conformational change and loss of activity. Interestingly, Scmidt et al., in 1986, carried out an enzymatic comparison between *Hymenoptera* venoms. In these findings, *Ectatomma tuberculatum* had low proteolytic activity of only 3 trypsin U/mg, according to a protocol established at the time, and *Ectatomma quadridens* venom did not show protease activity [54]. In the present work, we describe the *Ectatomma opaciventre* venom containing a high enzymatic activity as comparable to a purified metalloprotease from *Bothrops pauloensis* [22], revealing how potent the proteolytic fraction of this species is. Another class of enzymes was revealed in the venom when it was previously inhibited with Pepstatin A. Pepstatin A is a peptide inhibitor isolated from actinomycetes with specific action on the catalytic dyad of aspartate residues present in aspartic proteases [61].

#### 3.1.3. Phospholipases A_2_

Phospholipase A_2_ (PLA_2_) represents a family of enzymes that hydrolyze the sn-2 ester bond of phospholipids [62]. They are largely distributed enzymes in Hymenoptera and found in ant venoms; however, the *Tetramorium caespitium* venom does not have reported phospholipase activity [63], indicating that this is not a widely distributed enzyme in ant venoms. These enzymes play an important role in disrupting the lipid bilayer of cells leading to cell lysis, pore formation, and inflammation. In *Hymenoptera* venoms, these proteins are considered neurotoxic, cytotoxic, and potent allergens [64,65,66,67]. This is unlike snake phospholipases, as *Hymenoptera* phospholipases are not lethal to their prey, but they could exert a synergistic effect with toxic proteins leading to prey lethality [52,54]. We can find in the literature two isoforms of phospholipases isolated from *Vespa affins* wasp venom, Ves-A 1.01 and Ves-A 1.02. These enzymes exhibited high catalytic activity and thermal stability when heated. In addition, these enzymes showed paralytic activity in crickets, with 12.5 μg/g needed to immobilize 50% of the insects under study [68]. Furthermore, Sol i 1 is a phospholipase with allergenic properties from the venom of *Solenopsis invicta* and showing high cross-reactivity with IgE from patients sensitized with the bite of other venomous Hymenoptera [69].

*Ectatomma opaciventre* venom showed indirect phospholipase activity in agar containing egg yolk and erythrocytes. The analysis of the phospholipase A_2_ repertoire of nine ant species demonstrated a range of activity among ant venoms in promoting agar diffusion halos, generally as low performance, except for the *Pogonomyrmex baldius* species, which showed equivalent phospholipase activity to *Vespula squamosa* and *Vespula pensylvanica* venom [54]. In this work, the *E. tuberculatum* and *E. quadridens* venoms weakly dissipated a phospholipase activity [54] in counterpoint with our findings, in which the *E. opaciventre* venom showed high catalytic activity, presenting 60% of the activity of an acidic PLA_2_ isolated from *Bothrops pauloensis* [23], which demonstrated the presence of these enzymes in *E. opaciventre* crude venom.

#### 3.1.4. L-Amino Acid Oxidases (LAAOs)

LAAOs are flavoenzymes that can catalyze the oxidative deamination of L-amino acid to its α-ketoacid and release the production of hydrogen peroxide and ammonia. LAAOs are found in different types of organisms such as bacteria, fungi, and snake venoms [70,71]. *E. opaciventre* venom has a clear staining and strong activity of this enzyme when compared to Bp-LAAO, an isolated oxidase L-amino acid of *Bothrops pauloensis* [24]. According to this data, we can suggest that our work is the first report on the L-amino acid enzyme class found in hymenoptera venoms. In addition, the role of LAAOs in animal venoms is not fully understood; however, studies show that these enzymes have important biological activities, such as apoptotic, cytotoxic, bactericidal, and hemorrhagic [70,72,73,74]. These biotechnological and pharmaceutical applications of L-amino acid can be also explored by future *E. opaciventre* venom studies, opening a new and unexplored research aspect of toxinology.

### 3.2. Analysis of Hemostatic Effects by E. opaciventre Venom

Our data demonstrate that the venom is rich in enzymes, known as strong agents, capable of acting on the hemostatic system of prey. The venom of *E. opaciventre* showed a thrombolytic effect on fibrin red clot, however, it was not able to induce the coagulation and hydrolysis of fibrinogen chains. Collagen-induced platelet aggregation was also strongly inhibited by the venom and reinforced when there was a previous incubation of platelets with *E. opaciventre* venom, showing a partial inhibition of ADP and epinephrine. Our data suggest that *E. opaciventre* venom could mainly act on collagen receptors, such as αvβ3 integrin. However, more investigation is necessary to completely explain the action mechanism on platelets. In this context, some similar findings on animal toxins have already shown the venom action by this receptor, which supports our data [75,76].

Our results also suggest the presence of fibrin-specific thrombolytic agents or activators and inhibitors of platelet aggregation of fibrinolysis [77], which can interfere with the blood coagulation cascade. The fibrinogenolytic activity from ant venom remains unclear; *Odontomachus bauri* venom, for example, not only showed fibrinogenolytic activity on fibrinogen α and β chains, but also exerted strong coagulant activity in bovine plasma in about 15 s, when compared to the positive control demonstrating the high variability of effects and plasticity of ant venoms [45]. Usually, predatory ant diets are composed of insect larvae, arthropods, and annelids, which means less evolutionary function for hunting. The effects and meanings of the role of these toxins for prey catching remains unknown; the presence of enzymes with a hemostasis effect in the venom may have evolved into a defense mechanism against predators, as birds and small mammals have ants as the base of their food chain.

### 3.3. Biotechnological Potential of E. opaciventre Venom

The results demonstrate a good scenario for bioprospecting, which reveals this venom as an untapped rich source of protein compounds. Despite considerable advances in pharmacological and non-pharmacological treatments, cancer remains one of the most common causes of death around the world. When we consider different types of cancer, lung cancer grows at an incidence of 2% per year, registering around 1.7 million new cases [78,79]. Despite advances in new therapies, lung cancer remains a major challenge in the development of new antitumor agents, since resistance to chemotherapeutics develops during treatment, showing a low toxicity by the treatment and the selectivity of compounds by tumor cells [79].

In agreement with the biological characterization of *E. opaciventre* venom, we investigated the cytotoxic effects in lung tumor and non-tumor cell lines. The *E. opaciventre* venom showed a higher cytotoxic effect against the A549 cells (lung tumor cells). Otherwise, to BEAS-2B, a non-tumorigenic bronchus cell, the venom only showed cytotoxicity at the highest concentration (100 µg/mL), suggesting a selectivity ability of some venom components by tumor cell types. Similar effects were reported with the action of *Pachycondyla* *sennearensis* venom in MCF-7, a breast cancer line. In this report, *P. sennearensis* induced cell death by apoptosis, inhibiting cell proliferation and F-actin polymerization, through an IGF-1-independent pathway [80].

Another important bioprospecting potential is treating Leishmaniosis. Leishmaniosis is a neglected tropical disease, which has a therapeutic approach with low efficacy and high toxicity for patients. Therefore, new studies which are looking for potential molecules for the treatment of these diseases have gained prominence [81,82]. *Dinoponera quadriceps* venom has demonstrated antiparasitic effects by inducing cell death in the promastigotes of *L. amazonensis* [13]. In this context, our work addressed the cytotoxic profile of *E. opaciventre* venom in three species of *Lesihmania*. We evaluated the response in *Leishmania amazonensis* and *Leishmania brasiliensis*, responsible for the integumentary manifestation of the disease, and in *Lesihmania infantum*, which causes the visceral form of leishmaniasis. The venom of *E. opaciventre* showed the capacity to interfere with the viability of the promastigote forms of these parasites, showing higher cytotoxic action against *L. infatum* and *L. amazonensis*. Moreover, the promastigote form of *Leishmania brasiliensis* has been shown to be more resistant in vitro to cytotoxic components present in the venom. The IC_50_ value of *E. opaciventre* venom was lower than those already shown by *Dinoponera quadriceps* venom, even for *Leishmania braziliensis*, which was more resistant to *E. opaciventre* venom. In addition, our data were able to show a high selectivity of the venom for parasites, as well as demonstrate its low toxicity in macrophages isolated from mice.

The data generated confirm the *E. opaciventre* venom as a promising source for biomolecules with biotechnological applications in processes involving the hemostatic system, as well as helping to discover new therapies for diseases such as cancer and parasites.

## 4. Conclusions

This study reports the first investigation on the biochemical, enzymatic, and functional characteristics of the venom from the solitary foraging predatory ant species *Ectatomma opaciventre*. We also investigated its bioprospecting properties. This venom presented a great diversity of proteins and peptides, with these components showing high enzymatic activity. First, we reported the profile and preference of Hymenoptera venoms for hydrolysis of hyaluronidases, which indicate an unpublished presence of LAAOs in ant venom.

The venom action on hemostatic system demonstrates that it was able to act on primary hemostasis inhibiting platelet aggregation, mainly agonized by collagen. The proteases present in the venom demonstrated thrombolytic activity in red blood clots. In addition, the bioactive compounds present in the venom also demonstrated a high cytotoxic effect on tumor cell lines, in contrast to the low cytotoxicity in a non-tumorigenic cell line. A broad antiparasitic spectrum was also observed in the activity on parasites unicellular and multicellular. Thus, the *Ectatomma opaciventre* ant venom presents biomolecules with high potential for the development of new research tools or therapeutical drugs of high interest in different disease models. In summary, our work opened a new source to investigate the *Ectatomma opaciventre* venom components and their pharmacological application to design new therapy approaches for many diseases.

## 5. Materials and Methods

### 5.1. Venom Preparation

The ants were collected at Panga Ecological Station, a private protected area belonging to the Federal University of Uberlândia, located 30 km south of Uberlândia in the state of Minas Gerais, Brazil (19°10′ S and 48°24′ W), and immediately transferred to the laboratory. The venom gland was pinched out with entomological forceps and placed in a microtube with 7.5% acetonitrile and 0.05% trifluoracetic acid/distilled water. The glands were disrupted by cold ultrasonic bath and centrifuged at 320× *g* for 2 min. Membrane debris and empty venom sacs were discarded. Total protein was measured by the Bradford assay [38]. The samples were lyophilized and kept at −80 °C.

### 5.2. Biological Samples

The crude venom of *Bothrops pauloensis* and *Crotalus durrissus collilineatus* was obtained from specimens kept in the Animal Toxin Extraction Center Ltd.—(CETA serpentarium), Morungaba, Sao Paulo, Brazil. This serpentarium has proof of registration of IBAMA and renewable natural resources (n° 2087163). Toxins isolated from the crude venom of *Bothrops pauloensis* were generously assigned to the work by Prof. Dr. Veridiana de Melo Rodrigues from the Federal University of Uberlândia. The venom of *Tityus serrulatus* was kindly given by Prof. Dr. Eliane Candiani Arantes’ research group. The specimens were obtained from the region of Ribeirão Preto and were maintained in the serpentarium of the Faculdade de Medicina de Ribeirão Preto (FMRP-USP, University of São Paulo at School of Medicine of Ribeirão Preto), Brazil, in accordance with the guidelines of Ibama, Brazilian Institute of Environment. Bovine blood and fractions were obtained at the experimental farm of the Federal University of Uberlândia. All the experiments were conducted according to the standards recommended by the CEUA/UFU. The bone marrow-derived macrophages (BMDM) were collected from female BALB/c mice, supplied by the animal facility of the Federal University of Uberlândia (CBEA/UFU) under the registration protocol 096/15 (CEUA/UFU, approved in November 2015).

### 5.3. Polyacrylamide Gel Electrophoresis (SDS-PAGE)

Gels were performed, as described by Laemmli et al. 1970 [83], using a continuous buffering system. The following solutions were used in 12.5% and 21% polyacrylamide gel electrophoresis (*w*/*v*) with denaturing agents sodium *dodecyl* sulfate (SDS). Proteins were stained with a solution of Coomassie blue R-250 and peptides were stained with silver (Sigma-Aldrich, Saint Louis, MO, USA). Molecular size markers (MrS) (Thermo Scientific #26610, Waltham, MA, USA) and (Ultra-low Range Molecular M354, Sigma) were used in the appropriate electrophoretic run.

### 5.4. Enzymatic Activities

#### 5.4.1. Fibrinogenolytic Activity

Fibrinogenolytic activity was evaluated, as described by Rodrigues et al. (2000) [84], with some modifications. Briefly, samples of 50 μL of bovine fibrinogen (Sigma-Aldrich), 1.5 mg/mL, in phosphate buffered saline (PBS), pH 7.8, were incubated with different crude venom concentrations (0.5 μg; 1 μg; 2.5 μg; 5 μg; 10 μg) for 2 h at 37 °C. The reaction was stopped with 25 μL of 0.06 M Tris–HCl, pH 6.8, containing 10% (*v*/*v*) glycerol, 10% (*v*/*v*) β-mercaptoethanol, 2% (*w*/*v*) SDS, and 0.05% (*w*/*v*) bromophenol blue. The samples were then heated at 100 °C for 5 min and analyzed by 12.5% SDS-PAGE.

#### 5.4.2. Azocaseinolytic Activity

Enzymatic characterization of crude venom was initially achieved using azocasein as a substrate according to Gomes et al. (2011) [22], with modifications. Samples containing 800 µL of azocasein (Sigma-Aldrich) (1 mg/mL) in PBS were incubated for 30 min at 37 °C with 5 μg of crude venom. Next, 100 μL of 20% trichloroacetic acid (m/v) was added to each sample. They were incubated at room temperature for 30 min, and then centrifuged at 1856× *g* for 20 min. The absorbance of the supernatant was determined at 405 nm by a BioTek EL800 reader. One unit (U) of azocaseinolytic activity was defined as an increase of 0.01 absorbance units at 405 nm under standard assay conditions.

The effect of protease inhibitors, 10 mm 1,10-phenanthroline, phenylmethylsulfonyl fluoride (PMSF), ethylenediamine tetraacetic acid (EDTA), and divalent cations, 10 mM, (Ca^2+^, Mg^2+^, Zn^2+^, Cu^2+^, Hg^2+^ and Ba^2+^) on azocaseinolytic activity was evaluated by incubating 5 µg of crude venom for 1 h at 37 °C. The enzymatic assay was performed as described above. The positive control was used 5 μg of Bothropoidin, a metalloprotease from *Bothrops pauloensis* venom [22].

#### 5.4.3. Zymography

Zimography was carried out according to Cevallos et al. (1992) [46], based on the electrophoresis system described by Laemmli (1970) [28], with adaptations. In this method, hyaluronic acid (0.4 mg/mL) and gelatin (1.0 mg/mL) were incorporated into the gel matrix during polymerization process. The analyzed samples were not reduced by β-mercaptoethanol and no denaturation occurred by heating to 100 °C. After the electrophoresis, the gel was washed twice for 30 min at room temperature in 2.5% Triton X-100 (Sigma-Aldrich) to remove the SDS. The gels were stained with R-250 Coomassie blue. Gelatin and hyaluronan enzyme activity were detected as colorless bands in the otherwise blue gel.

#### 5.4.4. Hyaluronidase Activity

The hyaluronidase activity was determined quantitatively by turbidimetry through a modified version of the Di Ferrante method (1956) [85] and adapted to a 96-well flat-bottom microplate. The acetate buffer (200 mm sodium acetate, 150 mm NaCl pH 6.0), 10 μg hyaluronan, and different substrates, such as chondroitin-4 (Chondroitin sulphate C) dermatan and chondroitin-6-sulphate (Sigma, Marlborough, MA, USA) (0.5 mg/mL in water), were adjusted within the final reaction volume to 200 μL, with different concentrations of crude venom. The mixture was incubated for 30 min at 37 °C and the reaction was quenched with the addition of 100 μL of 5% cetyltrimethylammonium bromide (CTAB) (*w*/*v*) and 4% NaOH (*w*/*v*). The microplate was read within 10 min in a microplate reader (Multiskan GO Thermo Scientific, Waltham, MA, USA), using a wavelength of 400 nm. The turbidimetric reduction unit (UTR) will be expressed as the amount of enzyme required to hydrolyze 50% of the hyaluronic acid.

#### 5.4.5. Phospholipase Activity

The phospholipase assay was performed according to De Haas and Postema (1968) [86]. Phospholipase activity was measured using egg yolk as the substrate in the presence of 0.03 m sodium deoxycholate and 0.6 m CaCl_2_. The mixture was incubated with 5 µg of crude venom. Results were expressed as mEqNaOH/mg/min. The positive control was 5 μg of Bp-TxI-PLA_2_, from *Bothrops pauloensis*.

For the indirect Phospholipase activity, we used the method described by Schröder et al. (1971) [87]. Briefly, PBS with 1.0% agarose was added under heating, until a clear colloid (final volume 10 mL) was formed. After cooling, the agarose solution (±40 °C) washed bovine erythrocytes, 0.01 m CaCl_2_, egg yolk (in the ratio of 1:3 PBS), and 0.005% sodium azide (*w*/*v*); the resulting solution was poured into a petri dish (the plate was maintained at room temperature for 30 min). The samples were applied in holes in the gel at the desired concentrations (10 µg and 20 µg), at the final volume of 30 μL, followed by incubation at 37 °C for 24 h and subsequent measurement of the halos.

#### 5.4.6. L-Amino Acid Oxidase Assay

LAAO activity was measured by an adaptation of the method previously described by Ponnudurai et al. (1994) [88]. In this assay, the oxidative deamination of different L-amino acids produced hydrogen peroxide, which was reduced in the presence of horseradish peroxidase (HRP) by *o*-phenylenediamine to produce a colored oxidized product, which was spectrophotometrically monitored at 490 nm. Briefly, 5 µg of crude venom was incubated at 37 °C for 1 h with 195 µL of a solution containing 80 µg of *o*-phenylenediamine, 8 mg of L-amino acids, and 10 µL HRP (1 mg/mL) in 10 mm Tris–HCl buffer at pH 7.2. The reaction was stopped by the addition of 100 µL of 10% (*w*/*v*) citric acid. One unit (1 U) of enzyme activity was defined as the amount of enzyme able to produce 1 mmol of hydrogen peroxide/min, under the described conditions.

### 5.5. Biological Activities

#### 5.5.1. Coagulant Activity

Coagulant activity on bovine plasma was performed as described by Assakura et al. (1992) [89], with modifications. Different concentrations of the crude venom were prepared in a final volume of 50 μL in 0.06 M Tris-HCl buffer, pH 7.2. Then, 0.2 M calcium chloride was added to 150 μL of bovine plasma and incubated at 37 °C. The samples were kept under constant and gentle stirring. The time required to start the formation of the fibrin network was recorded by a photometric system in the coagulometer Quick Timer II (Draker–BR) and compared with the positive and negative control groups. Clotting time duration of more than 240 s was considered indicative of a non-coagulant sample. As control of the reaction, we used 50 μg of *Crotalus durrissus collilineatus* venom.

#### 5.5.2. Thrombolytic Activity

Thrombolytic activity was assessed by dissolving thrombi on a 24-well plate. Briefly, bovine blood collected in the absence of anticoagulants. Briefly, bovine blood was collected and 1 mL of blood per well was placed in the plate and incubated at 37 °C for spontaneous clotting. The clots obtained were treated with different amounts of venom (50 µg and 100 µg), adjusted to 500 μL of final solution with PBS for 48 h at 37 °C. For positive control 50 μg of *Bothrops pauloensis* venom was used and for negative control PBS was used. After treatment, the clots were measured and weighed to evaluate the activity.

#### 5.5.3. Platelet Aggregation Assay

Platelet aggregation assays were performed on bovine platelet-rich plasma (PRP) and measured using an automated 4-channel Aggregometer (AggRAM version 1.1, Helena Laboratories). Blood collected from bovines was placed in sodium citrate (3.2%, *w*/*v*) and centrifuged at 100× *g* for 12 min at room temperature to obtain PRP. Platelet-poor plasma (PPP) was obtained from the residue by centrifugation of citrated blood at 1000× *g* for 15 min, used for instrument calibration. Assays were carried out using 200 μL of PRP maintained at 37 °C under continuous stirring in siliconized glass cuvettes. Aggregation was triggered with collagen (10 μg/mL), adenosine diphosphate (ADP) (20 μM), or epinephrine (300 μM). Agonists were added concomitantly with the *E. opaciventre* venom in the platelet solution at the time of testing, or the platelets were pre-treated for 20 min with the crude venom. One hundred percent (100%) aggregation was expressed as the percentage absorbance relative to PPP aggregation. Control experiments were performed using only platelet agonists.

#### 5.5.4. Cell Viability Assay

##### Cell Culture

The human cell line BEAS-2B (CRL-9609), non-tumorigenic bronchus cell, and A549 (CRM-CCL-185), an adenocarcinoma human alveolar basal epithelial cell, were used. Cells were obtained from the American Type Culture Collection (ATCC; Virginia, USA). The BEAS-2B and A549 cells were grown in RPMI-1640 medium (Sigma), supplemented with 10% fetal bovine serum (FBS) (Cultilab) and containing 100 U/mL penicillin and 100 µg/mL streptomycin (Sigma). Cell cultures were incubated at 37 °C with 5% CO_2_.

##### MTT Assay

For the viability assays, the A549 and BEAS-2B cells were treated with crude venom following the methodology of Mosmann (1983) [90] and assessed by an MTT assay [3-(4,5-dimethylthiazol-2-yl)-2,5-diphenyltetrazolium bromide], with few modifications. Briefly, cells were seeded on 96-well plates at the density of 2 × 10^4^ cells/well followed by incubation for 24 h at 37 °C in a humidified incubator containing 5% CO_2_. After this period, cells were treated with different concentrations (two-fold serial dilution from 100 μg/mL to 0.048 μg/mL) of *E. opaciventre* crude venom for 24 h. After the treatment, 20 μL MTT per well (Sigma M2128, Marlborough, MA, USA) (5 mg/mL) was added and incubated for 3 h, and the formazan crystals were dissolved in 100 μL PBS with 10% SDS and 0.01 N HCl for 18 h until the complete dissolution of the crystals. Optical density was determined at 570 nm in a plate reader (Multiskan GO Thermo Scientific, Waltham, MA, USA). Viability was expressed as a percentage compared to the untreated control.

$$\mathrm{Viability}\;\left(\%\right)=\frac{{\mathrm{Abs}}_{570\;\mathrm{sample}}-{\mathrm{Abs}}_{570\;\mathrm{control}}}{{\mathrm{Abs}}_{570\;\mathrm{control}}}\times 100$$

#### 5.5.5. Antiparasitic Activity

##### Leishmania Promastigote Culture

*Leishmania (Leishmania) amazonensis* (IFLA/BR/67/PH8 strain), *Leishmania (Viannia.) braziliensis* (MHOM/BR/75/M2904 strain), and *Leishmania (Leishmania) infantum* (MCER/BR/79/M6445 strain) promastigotes were cultured in Liver Infusion Tryptose LIT medium, pH 7.4, supplemented with 10% FBS, penicillin (100 UI mL^−1^), and streptomycin (100 μg mL^−1^), in 2% glucose (*w*/*v*)—complete LIT—at 23 ± 0.5 °C. Promastigotes used in all experiments were isolated from the stationary growth phase. Promastigotes (5 × 10^5^ promastigote/well) were placed on 96-well culture plates and incubated at 23 °C with different concentrations of crude venom (two-fold serial dilution from 100 μg/mL to 0.048 μg/mL) for 72 h. The cell viabilities were evaluated by the MTT assay according to Mosmann (1983) [90], with some modifications; and the 50% inhibitory concentration (IC_50_) of venom on cell viability was then determined by GraphPad Prism 5.0 (GraphPad Software Inc., San Diego, CA, USA).

##### Evaluation of Susceptibility of Mouse Bone Marrow-Derived Macrophages (BMDM) Treated with *E. opaciventre* Venom

BMDM were obtained by differentiating bone marrow cells from female BALB/C mice (aged 7–9 weeks), as previously described [26] and approved by ethical statement 096/15 (CEUA/UFU). Briefly, in this method, cells obtained from the bone marrow are processed to remove red blood cells. After this step, cells are resuspended in RPMI medium containing 20% L929 supernatant and cultured for 7 to 8 days for cell differentiation. Then, macrophages (2 × 10^5^ cells/well) were plated in 96-well culture plates containing RPMI 1640 medium supplemented with 20% FBS, 1% penicillin (10,000 UI mL^−1^), and streptomycin (10 mg mL^−1^), in 1.3% glutamine (*w*/*v*)—complete RPMI medium—in an incubator containing 5% CO_2_ at 37 °C for 18 h. After, BMDM were incubated with a complete RPMI medium in the absence (control) or presence of different concentrations (two-fold serial dilution from 100 μg/mL to 0.78 μg/mL) of crude venom for 24 h. The cell viabilities were assessed by the MTT assay according to Mosmann, as described before [90].

### 5.6. Statistical Analysis

All assays were performed in triplicate. The statistical analysis of variance (ANOVA) and Student’s t-test, with a significance level of 5% (*p* < 0.05) represented by an asterisk signal, were performed using the software GraphPad Prism, version 9.0 (GraphPad Program Inc., San Diego, CA, USA).

## Figures and Tables

**Table 1:** L-amino acid oxidase performance. Activity of *E. opaciventre* on deamination of L-amino acid. 5 µg of crude venom incubated with different L-amino acid for 1 h. The reaction was stopped with acid citric 10% (*v*/*v*). One unit (1 U) of enzyme activity was defined as the amount of enzyme able to produce 1 mmol of hydrogen peroxide/min.

Amino Acids	*E. opaciventre*
Serine	384.18
Valine	310.43
Glutamine	290.05
Alanine	259.49
Arginine	161.48
Asparagine	153.2

## Data Availability

Data available in a publicly accessible repository that does not issue DOIs Publicly available datasets were analyzed in this study. This data can be found here: https://repositorio.ufu.br/?locale=pt_BR (accessed on23 December 2021).
